# Surgery and Radioactive Iodine Therapeutic Strategy for Patients Greater Than 60 Years of Age with Differentiated Thyroid Cancer

**DOI:** 10.1155/2022/4348396

**Published:** 2022-02-08

**Authors:** Tao Tang, Jingtai Zhi, Wei Zhang, Linfei Hu, Xianhui Ruan, Xiaoyu Chen, Zhaohui Wang, Xiangqian Zheng, Ming Gao

**Affiliations:** ^1^Department of Thyroid and Neck Tumor, Tianjin Medical University Cancer Institute and Hospital, National Clinical Research Center for Cancer, Key Laboratory of Cancer Prevention and Therapy, Tianjin's Clinical Research Center for Cancer, Tianjin, China; ^2^The First Department of Hepatobiliary Surgery, Affiliated Hospital of North Sichuan Medical College, Institute of Hepato-Biliary-Pancreatic-Intestinal Disease, North Sichuan Medical College, Nanchong, China; ^3^Department of Otolaryngology Head and Neck Surgery, Tianjin First Central Hospital, Tianjin, China; ^4^Department of Head and Neck Surgery, Sichuan Cancer Hospital and Institute, Sichuan Cancer Center, School of Medicine, University of Electronic Science and Technology of China, China; ^5^Department of Breast and Thyroid Surgery, Tianjin Union Medical Center, Tianjin, China

## Abstract

Purpose: The purpose of the current study was to determine whether older patients with differentiated thyroid cancer (DTC) who received surgical treatment had a better cause-specific survival (CSS) than patients who were recommended surgery, but declined, and whether patients who underwent postoperative RAI-131 therapy had an impact on CSS based on TNM staging and number of lymph node metastases for all total or near-total thyroidectomy patients. *Patients and Methods*: This retrospective, population-based study analyzed the clinical data of 162 DTC patients from signal institution in China and 26,487 cases from the Surveillance, Epidemiology, and End Results (SEER) program registry. The patients were divided into two groups (underwent surgery and surgery recommended, but not performed) in the SEER cohort. Furthermore, patients were grouped as follows: T4; N1b; M1; T1-3N0-1a; specific number of lymph node metastases; and total or near-total thyroidectomy. *Results*: The 120-month cause-specific survival (CSS) rate of women and men showed a gradual declining trend from 60–64 to ≥80 years of age in the group that underwent surgery. The CSS rate of women and men showed a marked downward and irregular trend with an increase in age in the recommended, but no surgery group in the SEER cohort. Univariate analysis indicated that the surgery group had a higher 120-month CSS in women in most stages and men, compared with the no surgery group in the SEER cohort. The analysis of the SEER cohort showed that RAI-131 therapy was associated with an improved 80-month CSS in T4/N1b/M1 women (*P* < 0.0183) and men (*P* < 0.0011). However, there were no CSS differences between the RAI-131 therapy and the no-RAI-131 group for the patients with T4/N1b/M1 (AJCC 7th) thyroid cancer in the Chinese cohort. There was no CSS difference in women or men between the T1-3N0 and T1-3N1a patients in the SEER cohort. And similar findings were observed in T1-3N1a patients in the Chinese cohort. There was no statistical difference between the two subgroups. *Conclusions*: Surgical treatment should be recommended for elderly DTC patients because surgery can lead to a better CSS. High-risk patients achieve a higher benefit-to-risk ratio with RAI-131 therapy. To avoid the adverse effects associated with RAI-131 therapy, a multidisciplinary discussion should be arranged for intermediate- and low-risk patients.

## 1. Introduction

Thyroid cancer is the most frequent endocrine malignancy and the incidence has nearly tripled in the past few decades in the US [1]. Among thyroid cancers, >90% are differentiated thyroid cancer (DTC), 5% are poorly differentiated thyroid cancer (PDTC), 1% are anaplastic thyroid cancer, and <3% are medullary thyroid carcinoma [2]. The incidence of thyroid cancer is approximately 4-fold higher in women than men. The significant increase in the incidence of thyroid cancer is ongoing and resulted in thyroid cancer becoming the third most common cancer in women of all ages by 2019 [3]. With the gradual increase in the elderly population and the popularization of physical examinations, more and more elderly patients with DTC have been diagnosed.

Surgery is the preferred treatment for most patients with differentiated thyroid cancer (DTC), which plays an important role in the prognosis of the disease. Previous studies showed that the strategy of total thyroidectomy with subsequent levothyroxine treatment could decreased the risk of both recurrence and mortality in patients with high-risk DTC without initial distant metastases [4]. In this study, we determined whether older thyroid cancer patients who underwent surgical treatment had a better cause-specific survival (CSS) than patients who were recommended to undergo surgery, but declined, without any pre-existing assumption or bias.

Postoperative disease status should be taken into consideration when deciding whether to recommend additional treatment. In general, postoperative radioactive iodine-131 (RAI-131) adjuvant therapy is routinely recommended for DTC patients at high risk based on the American Thyroid Association (ATA) guidelines. RAI-131 therapy has been used for patients for the following indications: remnant ablation; potential micrometastases; locoregional invasion; and pulmonary or bone metastatic disease. The purpose of therapy is to ensure a tumoricidal effect [5, 6]. Treatment has been recommended more selectively in recent years as guidelines have evolved to reflect risks and utility in patient subsets. However, is treatment suitable for all patients with lymph nodes metastases and can treatment reduce the risk of death or recurrence?

We sought to determine the outcomes between patients who underwent surgery and patients in whom surgery was recommended but declined. We also determined the outcomes between elderly patients with DTC who did and did not receive postoperative RAI-131 therapy.

## 2. Patients and Methods

### 2.1. Patients

#### 2.1.1. Surveillance, Epidemiology, and End Results (SEER) Database

The SEER data were derived from the SEER database (November 2016 submission) using SEER*∗*Stat software (National Cancer Institute). All data regarding demographic characteristics and cancer incidence were obtained from the SEER*∗*Stat 8.3.6 program of incident cases from 1975–2016 (November 2018 submission) [7]. DTC cases between 2004 and 2011 in the SEER public access database and their corresponding details were retrieved [7]. Patients with incomplete or missing information were excluded. SEER is the primary source for cancer statistics in the United States and represents 28% of the US population [8]. SEER is considered as representative of the US in terms of demographic composition, as well as cancer incidence and mortality rate.

**Figure 1:**
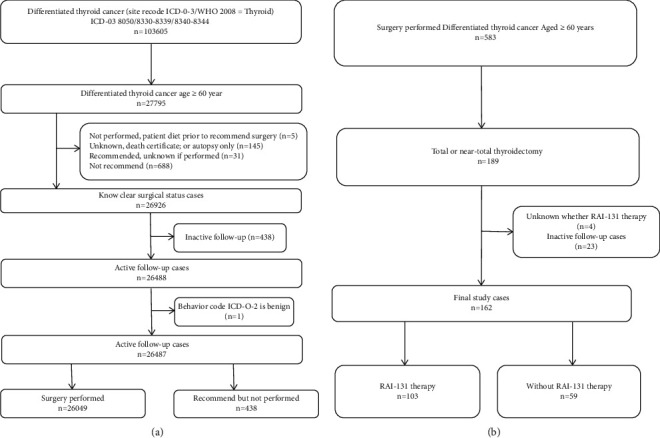
Exclusion criteria utilized to derive the final study cohort from the SEER database and Chinese database. (a). SEER database final study cohort; (b). Chinese database final study cohort.

Patients who met the following criteria were selected (Figure 1). Only patients aged older than 60 years were considered. The study was granted exempt status by our Institutional Review Board. Moreover, the following patients were excluded: died prior to the recommendation for surgery; diagnosed with DTC at the time of autopsy or on the death certificate only were recommended to undergo surgery but it was unknown if surgery was performed; had incomplete follow-up evaluations; and were not recommended to undergo surgery. The enrolled patients were divided into two groups (surgery performed and surgery recommended, but not performed).

Furthermore, we also aimed to identify whether postoperative RAI-131 therapy had an impact on CSS with different TNM stage and the number of positive lymph nodes. Thus, we divided patients with T4, N1b, M1, T1-3N0-1a, and the number of metastatic lymph nodes who underwent a total or near-total thyroidectomy.

#### 2.1.2. Chinese Database

We retrospectively collected the clinical data of 583 DTC patients who received thyroidectomy at Sichuan Cancer Hospital between January 1, 2000, and September 31, 2011. All procedures were followed by the ethical standards of the responsible committee on human experimentation (institutional and national) and with the Helsinki Declaration of 1964 and later versions. All patients received standard follow-up, including laboratory and clinical examinations after discharge from the hospital every 3 months for the first 3 years, every 6 months during the fourth and fifth years, and once a year thereafter until the patient died or until the date of last follow-up (September 2020). Three doctors in each medical center are responsible for follow-up and recording of patients' information.

### 2.2. Outcome Measures

The outcomes of interest included thyroid CSS and duration of survival for men and women. Duration of survival was defined as the time from the date of diagnosis to the date of death or last contact. Cause of death was defined using the SEER cause of death recode. Patients who died not due to thyroid carcinoma were designated as CSS while patients diagnosed with thyroid carcinoma who were still alive as overall survival.

### 2.3. Statistical Analysis

Mean and interquartile ranges were reported for continuous variables. Frequency and proportion were reported for categorical variables. Pearson *χ*^2^ test or Fisher's exact test were used to compare means and proportions, respectively, between categorical variables and treatment groups. The Kaplan Meier estimator was used to determine thyroid cancer CSS and plot survival curves. Cox proportional hazards regression was used to determine hazard ratios (HRs) with 95% confidence intervals (CIs). All statistical tests were two-sided and statistical significance was defined as a *P* < 0.05.

## 3. Results

### 3.1. Patient Characteristics

Overall, in the SEER cohort, 26,487 elderly patients (≥60 years of age) diagnosed with DTC were identified; 26,049 patients underwent surgical treatment and 438 were recommended to undergo surgery, but surgery was not performed. The mean age at the time of diagnosis was 73.50 (SD ± 7.07) years, 68.97 (SD ± 6.90) years in the group that had surgery, and 76 (SD ± 8.64) years in the group that was recommended to have surgery but did not undergo surgery (Table 1). After applying the inclusion and exclusion criteria, 162 DTC patients who received thyroidectomy in Sichuan Cancer Hospital were analyzed. And in the Chinese cohort, the mean age at the time of diagnosis was 73.50 (SD ± 7.07) years and 68.97 (SD ± 6.90) years in the group that had surgery (Table 1).

In the surgery group of SEER cohort, 463 and 200 T4/N1b/M1 (7^th^ American Joint Committee on Cancer (AJCC)) patients did and did not receive RAI-131 therapy, respectively. Of 2548 patients, 223 (not T1-3N0) underwent total or subtotal thyroidectomy and 209 and 132 T1-3N1a patients underwent total or subtotal thyroidectomy, respectively. In these cohorts, the mean ages were 69.52 (SD ± 7.18) versus 71.87 (SD ± 8.25) years, 67.90 (SD ± 6.11) versus 67.91 (SD ± 6.36) years, and 67.88 (SD ± 6.64) versus 67.77 (SD ± 6.54) years between patients who did or did not receive RAI-131 therapy, respectively (Table 2).

In the Chinese cohort, 103 patients received RAI-131 therapy, while 59 patients did not receive RAI-131 therapy. The characteristic of these was shown in Table 2.

### 3.2. Survival Outcomes

The 120-month CSS rate of women (96.27%–81.92%) and men (95.54%–79.86%) showed a gradual declining trend from patients 60–64 to ≥ 80 years of age and stage in the group that had surgery in the SEER cohort. The CSS rate of women (90.68%–52.00%) and men (94.74%–16.22%) showed a marked downward and irregular trend with an increased age in the group recommended to have surgery, but who did not have surgery. And in the Chinese cohort, the 120-month CSS rate of women (92.86%–69.33%) and men (92.31%–71.59%) also showed a gradual declining trend from patients aged 60–64 to patients aged older than 80.

**Figure 2:**
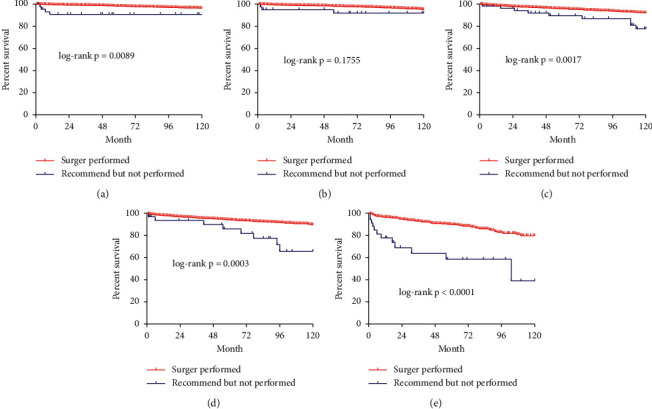
Male Kaplan–Meier survival curve of cause-specific survival comparing the surgery group with the group recommended to have surgery but did not have surgery of different ages and stages. (a). 60–64 years of age; (b) 65–69 years of age; (c). 70–74 years of age; (d). 75–79 years of age; (e). 80–84 years of age; (f). ≥ 85 years of age.

**Figure 3:**
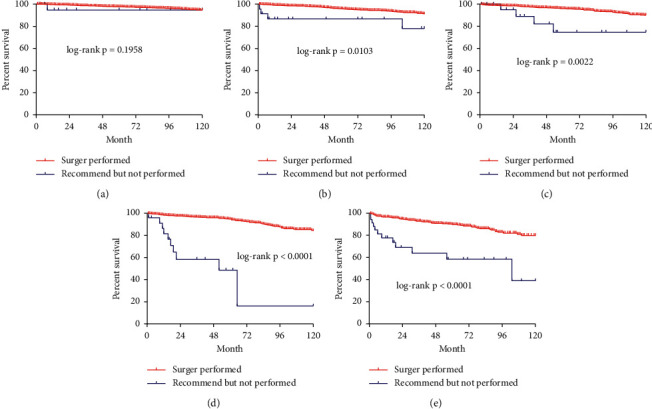
Female Kaplan–Meier survival curve of cause-specific survival comparing the surgery group with the group recommended to have surgery but did not have surgery of different ages and stages. (a). 60–64 years of age; (b) 65–69 years of age; (c). 70–74 years of age; (d). 75–79 years of age; (e). 80–84 years of age; (f). ≥ 85 years of age.

Univariate analysis indicated that the surgery group in the SEER cohort had a higher 120-month CSS among women in most stages and men, compared with the group that did not have surgery (Figures 2 and 3).

**Figure 4:**
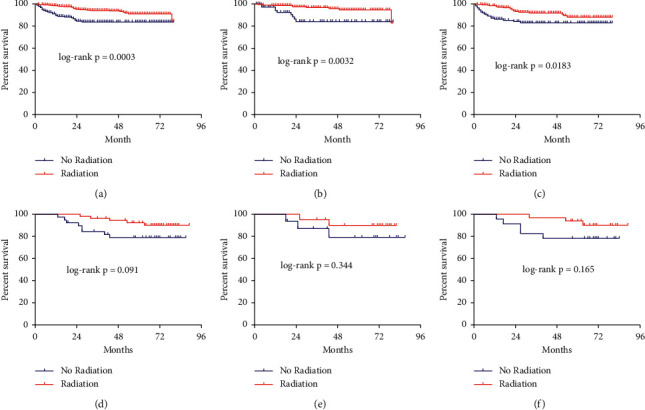
Kaplan–Meier survival curve of cause-specific survival comparing undergone RAI-131 therapy with not undergone of high-risk patients. (a). The total patients of T4/N1b/M1 (AJCC 7th) in SEER cohort. (b). The male patients of T4/N1b/M1 (AJCC 7th) in SEER cohort. (c). The female patients of T4/N1b/M1 (AJCC 7th) in SEER cohort. (d). The total patients of T4/N1b/M1 (AJCC 7th) in Chinese cohort. (e). The male patients of T4/N1b/M1 (AJCC 7th) in Chinese cohort. (f). The female patients of T4/N1b/M1 (AJCC 7th) in Chinese cohort.

**Figure 5:**
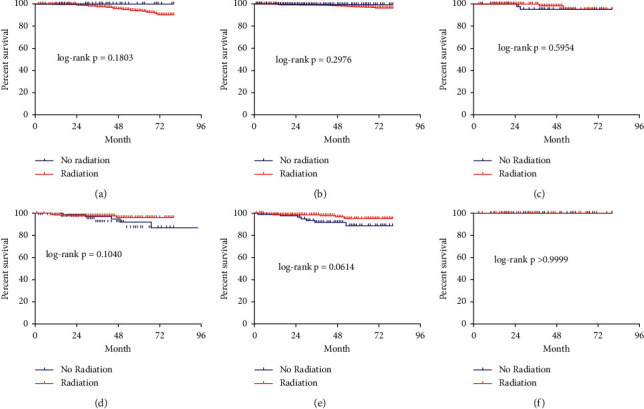
Kaplan–Meier survival curve of cause-specific survival comparing T1-3N0-1a (7th AJCC) patients who did and did not receive RAI-131 therapy; (a). T1-3N0 male patients; (b). T1-3N0 female patients; (c). T1-3N1a male patients; (d). T1-3N1a female patients; (e). T1-3N1a patients with 1–3 lymph node metastases; (f). T1-3N1a patients with >3 lymph node metastases.

RAI-131 therapy was associated with an improved 80-month CSS in T4/N1b/M1 (AJCC 7th) women (HR: 0.47, 95% CI: 0.24–0.95, *P* < 0.018) and men (HR: 0.27, 95% CI: 0.09–0.84, *P* < 0.0011; Figure 4(a)–4(c)) in the SEER cohort. There were no differences in CSS among T1-3N0 (*P*=0.2976, *P*=0.1650) and T1-3N1a female and male patients (*P*=0.1040, *P*=0.5954; Figures 5(a)–5(d)). Furthermore, we also investigated whether the specific number of positive lymph nodes (PLNs) in T1-3N1a patients had different effects on the 80-month CSS, so we divided these patients into subgroups with 1–3 and >3 PLNs; there was no statistical significance between the two subgroups (*P*=0.0614, *P* > 0.9999; Figures 5(e) and 5(f)).

However, in the Chinese cohort, there are no differences between the RAI-131 therapy and the no-RAI-131 group for the patients with T4/N1b/M1 (AJCC 7th) thyroid cancer (HR: 0.381, 95% CI: 0.124–1.17, *P*=0.091; Figure 4(d)). Similar findings were observed in patients in the Chinese cohort stratified by women (HR: 0.363, 95% CI: 0.872–1.154, *P*=0.165; Figure 4(e)) and men (HR: 0.4176, 95% CI: 0.068–2.54, *P*=0.344; Figure 4(f)). And there were no differences in CSS among T1-3N1a female (*P*=0.9904, Supplementary Figure 1A) and male patients (*P*=0.6640; Supplementary Figure 1B).

## 4. Discussion

Considerable changes have occurred in the management of differentiated thyroid cancer (DTC) during the past four decades, based on improved knowledge of the biology of DTC and on advances in therapy, including surgery, the use of radioactive iodine (radioiodine), thyroid hormone treatment, and availability of recombinant human TSH [4]. Surgery is the preferred treatment for most patients with differentiated thyroid cancer (DTC). DTC is the only human malignancy to include age as a part of the AJCC staging system, with a distinct cut point at 55 years of age [9]. Older age was also associated with a greater probability of a death from other reasons. Therefore, the benefit and risk of surgery should be considered as part of a balanced clinical approach due to the possibility of death from both DTC and other causes in older patients. It is empirically clear that many older patients, especially patients with smaller thyroid cancers, do not progress and if carefully triaged are otherwise eligible for monitoring. The individual acceptance of risk (from cancer or from surgery) thus remains a matter of informed consensus between patients and clinician [10, 11]. The aim of our study was to investigate the prognostic differences between older patients who underwent surgery and patients in whom surgery was recommended but declined in order for better treatment selection of elderly DTC patients.

Age has been identified as a well-recognized prognostic determinant of CSS in older patients, although DTC is relatively indolent. Age has a linear dose-dependent relationship with the DTC mortality rate. An age-specific cutoff corresponding to a marked decrement in survival is not apparent. Therefore, DTC patients have a higher cancer-specific mortality rate in elderly patients, rather than the tumor becoming more inactive as age increases [12, 13]. Another reason that DTC-related mortality accounts for only a fraction of the overall deaths among patients who underwent surgery is that comorbidities increased overall mortality by increasing the probability of other causes of mortality. Indeed, patients died earlier from other comorbidities and had a low probability of mortality from DTC. The roles of comorbidities and mortality from other causes were more pronounced in patients with a lower stage of DTC [14–16]. To eliminate these distractions, we only explored the CSS of patients from the SEER database and a single Chinese database. In the current study, we concluded that older DTC patients, especially patients aged older than 70 years who underwent thyroidectomy, had a better 10-year CSS. Such patients treated with surgery acquired a longer survival time in the case that there were no additional high-risk diseases associated with death. In fact, some patients were not recommended to undergo surgery because of other diseases or complications. In the current study, the patients in the SEER database recommended to undergo surgery, but who declined surgery were identified as the no surgery group and the effect of other diseases and complications were excluded. Therefore, the results were more favorable for the treatment strategy of elderly patients with DTC.

The 2015 ATA guidelines suggest modifications in risk stratification for DTC patients. The consensus guidelines recommend that RAI-131 should be offered to high-risk patients to reduce the risk of disease recurrence or mortality for DTC patients after a total or near-total thyroidectomy [17]; however, inappropriate use of RAI places patients at unnecessary risk of permanent treatment-related toxicity and secondary cancers [18]. Postoperative adjuvant RAI therapy has become more selective based on current guidelines [5, 19].

The updated guidelines have supported both decreased RAI doses for select populations, as well as expanded definitions for low- and intermediate-risk patients that may not require RAI-131. A growing body of literature indicates that the benefit-to-risk ratio is necessary to accurately identify patients who are ideal candidates for the therapy. RAI-131 can avoid unnecessary treatment, cost, and adverse effects at the same time [5, 17, 20, 21]. Evidence surrounding the use of RAI-131 in patients with intermediate-risk DTC is less robust and there is no consensus regarding the benefit-to-risk ratio [22].

The debate centers on the question of appropriate use of postoperative adjuvant RAI therapy. T4/N1b/M1 (7^th^ AJCC) patients who received RAI-131 therapy had a better CSS than patients who did not receive RAI-131 therapy. However, there was no difference in the number of PLNs (0, 1–3, and >3) among T1-3N0-1a patients (7^th^ AJCC), which are stage I or II (8^th^ AJCC) in the SEER cohort. There was no difference in T1-3N0-1a patients with 0, 1–3, and >3 PLNs. And the similar finding was observed in the T1-3N1a DTC patients in the Chinese cohort. Therefore, the patients at high risk have a higher benefit-to-risk ratio by RAI-131 therapy. Otherwise, the intermediate- or low-risk patients have fewer benefits from RAI therapy, which is similar with the patients only stimulating hormone-suppressive therapy and without RAI-131 therapy. And the patients who did not receive RAI therapy could avoid the associated risks of adverse effects.

The endocrinologist and nuclear medicine physician have an indispensable role in RAI-131 decision-making, the role of surgeons has been shown to exert substantial indirect influence on RAI-131 use, and the treatment concept is often a pivotal factor in postoperative management, especially for intermediate- and low-risk patients. Such patterns, however, may lead to inappropriate use of RAI-131. The main aim of this study was to selectively leverage the strengths of RAI-131 therapy and determine whether use of RAI-131 is suitable for high-risk patients who benefit, as well as intermediate- and lower-risk patients who may not benefit based on a multidisciplinary, risk-adapted approach [23].

There were several limitations to the current study. This was a nonrandomized observational study with the possibility of selection bias and inherent coding errors, especially an inability to confirm histological status in patients who did not undergo surgery. The SEER database did not capture key information variables, such as the largest size of metastatic lymph nodes, extranodal extension, vascular invasion, the histologic subtype, genetic mutations, specific RAI-131 dose, and other tumor or nodal factors that may have influenced on the decision to administer RAI-131 treatment, cancer recurrence, and prognosis outcomes. The number of patients recommended to undergo surgery, but who did not have surgery was small, and the TNM status of most patients was blank, so we cannot further analyze the influence of different TNM staging on CSS.

In conclusion, our analysis indicated that surgical treatment should be recommended to elderly DTC patients because elderly DTC patients treated with surgery can achieve a better CSS. The high-risk patients have a greater benefit-to-risk ratio through RAI-131 therapy. To avoid the related adverse effects associated with RAI-131 therapy, a multidisciplinary discussion about treatment options depending on the balance between clinical benefit and treatment-related toxicity should take place for intermediate- and low-risk patients.

## Figures and Tables

**Table 1:** Baseline demographic and clinical features of total patients in this study.

Characteristics	SEER cohort		Chinese cohort
	Surgery performed (*n* = 26049)	Recommended but not surgery (*n* = 438)	Surgery performed (*n* = 162)
Age at diagnosis (mean ± SD)	68.98 ± 6.90	73.56 ± 8.64	69.71 ± 7.72

60–64 years	8273(31.76%)	82(18.72%)	56(34.57%)
65–69 years	7211(27.68%)	72(16.44%)	50(30.86%)
70–74 years	4979(19.11%)	91(20.71%)	20(12.35%)
75–79 years	3300(12.67%)	70(15.98%)	13(8.02%)
80–85 years	1606(6.17%)	65(14.84%)	9(5.56%)
85+years	680(2.61%)	58(13.24%)	4(2.47%)
Sex			
Male	7987(30.66%)	149(34.02%)	53(32.72%)
Female	18062(69.37%)	289(65.98%)	99(61.11%)
Race			
Black	1987(7.63%)	29(6.62%)	—
White	21606(82.94%)	349(79.68%)	—
Other	2316(8.89%)	53(12.10%)	—
Unknown	140(0.53%)	7(1.60%)	—
Surgery extent			
Surgery	26049(100%)	0(0.00%)	162(100%)
No surgery	0(0.00%)	438(100%)	0

SD, standard deviation.

**Table 2:** Different TNM stage patients of received RAI-131 therapy.

Characteristics	SEER cohort		Chinese cohort	
	RAI-131 therapy	Not RAI-131 therapy	RAI-131 therapy	Not RAI-131 therapy
(AJCC 7th)	(*n* = 3220)	(*n* = 555)	(*n* = 103)	(*n* = 59)

Age of T4/N1b/M1 (mean ± SD)	69.52 ± 7.18	71.87 ± 8.25	69.66 ± 7.27	72.42 ± 8.58
Male	215(6.68%)	82(14.77%)	21 (20.39%)	16 (27.12%)
Female	248(7.70%)	118(21.26%)	33 (32.03%)	23(38.98%)

Age of T1-3N0 (mean ± SD)	67.90 ± 6.11	67.91 ± 6.36	—	—
Male	793(24.63%)	50(9.01%)	—	—
Female	1755(54.50%)	173(31.17%)	—	—

Age of T1-3N1a (mean ± SD)	67.88 ± 6.64	67.77 ± 6.54	67.89 ± 7.18	70.33 ± 7.15
Male	76(2.36%)	59(10.63%)	19 (18.45%)	7(11.86%)
Female	133(4.13)	73(13.15%)	30 (19.74%)	13(22.03%)

SD, standard deviation.

## Data Availability

The SEER data were derived from the SEER database (November 2016 submission) using SEER*∗*Stat software (National Cancer Institute).

## Supplementary Materials

Supplementary Materials
*Supplementary Figure 1*. Kaplan–Meier survival curve of cause-specific survival comparing undergone RAI-131 therapy with not undergone of T1-3N1a patients (AJCC 7th) in Chinese cohort. (A). The male patients of T1-3N1. (B). The female patients of T1-3N1a ().
