# Supplementary figures and images for: Surgery and Radioactive Iodine Therapeutic Strategy for Patients Greater Than 60 Years of Age with Differentiated Thyroid Cancer
Source: J Healthc Eng. 2022 Feb 8;2022:4348396. doi: 10.1155/2022/4348396 (PMC8846970; doi:10.1155/2022/4348396)

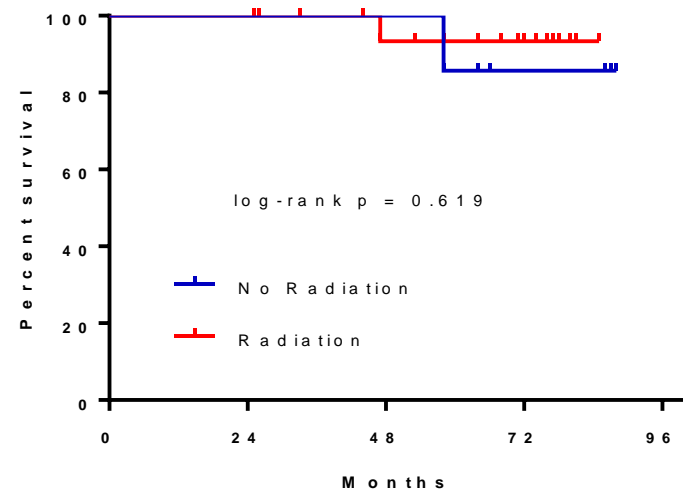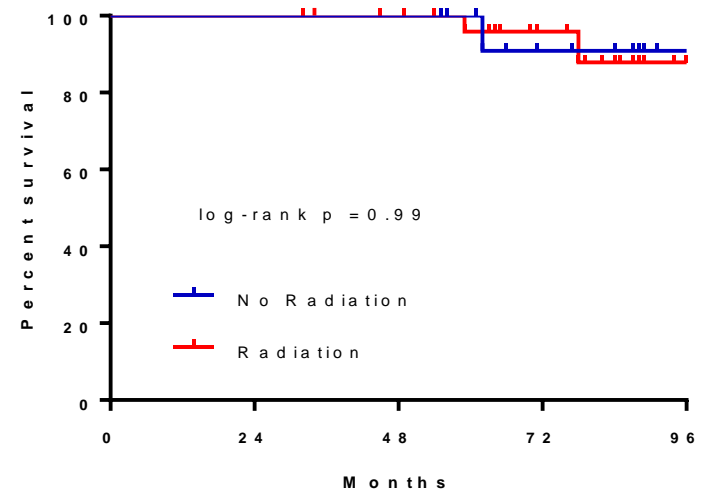

Supplement: Supplementary Materials — Supplementary Figure 1. Kaplan–Meier survival curve of cause-specific survival comparing undergone RAI-131 therapy with not undergone of T1-3N1a patients (AJCC 7th) in Chinese cohort. (A). The male patients of T1-3N1. (B). The female patients of T1-3N1a (). [file 4348396.f1.pdf]
